# Co-Treatments of Edible Curcumin from Turmeric Rhizomes and Chemotherapeutic Drugs on Cytotoxicity and FLT3 Protein Expression in Leukemic Stem Cells

**DOI:** 10.3390/molecules26195785

**Published:** 2021-09-24

**Authors:** Fah Chueahongthong, Singkome Tima, Sawitree Chiampanichayakul, Cory Berkland, Songyot Anuchapreeda

**Affiliations:** 1 Division of Clinical Microscopy, Department of Medical Technology, Faculty of Associated Medical Sciences, Chiang Mai University, Chiang Mai 50200, Thailand; fahmyfah@hotmail.com (F.C.); singkome@gmail.com (S.T.); chiampanich@gmail.com (S.C.); 2 Cancer Research Unit of Associated Medical Sciences (AMS-CRU), Faculty of Associated Medical Sciences, Chiang Mai University, Chiang Mai 50200, Thailand; 3 Center for Research and Development of Natural Products for Health, Chiang Mai University, Chiang Mai 50200, Thailand; 4 Department of Pharmaceutical Chemistry, School of Pharmacy, University of Kansas, Lawrence, KS 66047, USA

**Keywords:** leukemia, leukemic stem cell, FLT-3, chemotherapeutic drug, curcumin, co-treatment

## Abstract

This study aims to enhance efficacy and reduce toxicity of the combination treatment of a drug and curcumin (Cur) on leukemic stem cell and leukemic cell lines, including KG-1a and KG-1 (FLT3^+^ LSCs), EoL-1 (FLT3^+^ LCs), and U937 (FLT3^−^ LCs). The cytotoxicity of co-treatments of doxorubicin (Dox) or idarubicin (Ida) at concentrations of the IC_10_–IC_80_ values and each concentration of Cur at the IC_20_, IC_30_, IC_40_, and IC_50_ values (conditions 1, 2, 3, and 4) was determined by MTT assays. Dox–Cur increased cytotoxicity in leukemic cells. Dox–Cur co-treatment showed additive and synergistic effects in several conditions. The effect of this co-treatment on FLT3 expression in KG-1a, KG-1, and EoL-1 cells was examined by Western blotting. Dox–Cur decreased FLT3 protein levels and total cell numbers in all the cell lines in a dose-dependent manner. In summary, this study exhibits a novel report of Dox–Cur co-treatment in both enhancing cytotoxicity of Dox and inhibiting cell proliferation via FLT3 protein expression in leukemia stem cells and leukemic cells. This is the option of leukemia treatment with reducing side effects of chemotherapeutic drugs to leukemia patients.

## 1. Introduction

Leukemia is among the top 10 cancers diagnosed globally. It is a group of cancers of early blood-forming cells, which are characterized by the uncontrolled production and accumulation of blast or immature abnormal blood cells in the peripheral blood and bone marrow. Leukemia can be divided into four major types according to the stage and cell of origin: acute myeloid leukemia (AML), acute lymphoid leukemia (ALL), chronic myeloid leukemia (CML), and chronic lymphocytic leukemia (CLL). AML is the most common type of acute leukemia in adults, with the highest incidence and death rate in both sexes. It can be distinguished by clonal expansion of abnormal myeloid blasts in bone marrow, peripheral blood, or other tissues. According to recent data, 15–25% of AML patients fail to achieve complete remission (CR) due to chemotherapy resistance and may show relapse, with the overall 5-year survival rate of approximately 40% [1,2]. Moreover, between 10 and 40% of newly diagnosed AML patients do not achieve CR with intensive induction therapy, and such patients are categorized as primary refractory or resistant [3]. Hence, AML is defined as an aggressive malignant myeloid disorder.

One theory of resistance and relapse in AML patients involves the presence of subpopulations of leukemic stem cells (LSCs) [4]. LSCs have been defined as human AML-initiating cell with a self-renewal capacity and the ability to give rise to heterogeneous lineages of cancer cells [2,5]. They can be identified by the cell surface phenotype CD34^+^ hematopoietic stem cell and CD38^−^ subpopulation (i.e., CD34^+^CD38^−^ cells) [6].

Traditional chemotherapeutic drugs are incapable of clearing the LSC population due to many reasons. First, these drugs have been designed to eliminate fast-dividing cells by inhibiting cell cycle progression [7]; thus, they are less effective against LSCs, which have a prolonged G_0_ phase of the cell cycle [8]. Second, the expression of P-glycoprotein (MDR1), a multidrug resistance efflux pump protein in LSCs, potentially removes cytotoxic agents from cancer cells [9]. In addition, LSCs can undergo mutations and epigenetic changes, creating resistance to conventional chemotherapy toxicity [10,11]. Thus, LSCs are thought to play a fundamental role in AML pathogenesis and have become a focal point for targeted AML therapies.

Although drug resistance in AML patients is common, the traditional chemotherapy remains a popular method for leukemia treatment since these drugs can access cancer cells that have spread throughout the body. Anthracycline antibiotics such as doxorubicin (Dox (14-hydroxydaunorubicin)) and idarubicin (Ida (4-demethoxydaunorubicin)) are generally used as standard chemotherapeutic agents for AML treatment [12]. Ida is normally prepared in a 1 mg/mL solution (sterile water or normal saline). For patients with AML, the recommended intravenous dose of idarubicin for induction therapy is 12 mg/m^2^ daily for 3 days by slow (10 to 15 min) infusion, while Dox is recommended to be used with a dose 50 mg/m^2^ daily for 3 days [13,14,15]. These drugs function by inhibiting topoisomerase II activity in DNA transcription and also trigger apoptosis or autophagy in cells [16]. The combination of anthracyclines and cytarabine in the initial treatment is capable of inducing complete remission (CR) in approximately 45–70% of patients [17]; however, more than 40% of CR cases eventually experience relapse within 2 years [18]. The previous studies on AML leukemic stem cells demonstrated that anthracycline is less effective in killing LSCs (CD34^+^/CD38^−^ cells) than committed leukemic cells (CD34^+^/CD38^+^ cells) [19], and the co-treatment of cytarabine and anthracyclines is less effective against primitive AML cells than against leukemia blasts [20,21]. Furthermore, with high dose administration, anthracyclines cause side effects in patients including nausea, vomiting, hair loss, and myelosuppression [22]. Several reports expressed their concern about the presence of cardiac, renal, and liver toxicity in patients treated with Dox [23,24]. Thus, combination therapy with natural substances exhibiting chemosensitizing and chemoprotective activities may be a promising strategy to overcome LSCs and reduce the side effects of anthracyclines.

Curcumin (Cur) is a natural polyphenol constituent of turmeric (*Curcuma longa* Linn.). It exhibits a wide range of pharmacological activities, such as antioxidant, anti-cancer, anti-inflammatory, and antimicrobial effects [25,26,27]. Previous studies reported that Cur exhibited a potent cytotoxic effect, induced cell death in several types of leukemic cell lines [28,29,30,31], and showed inhibitory effects on WT1 and FLT3 protein expression, which are associated with cell proliferation [29,32,33]. Moreover, Cur inhibited the activity of P-glycoprotein (MDR1) [34] and exhibited cancer chemopreventive properties, especially in myocardial protection [35] by inhibiting ROS generation [36]. Consequently, it may be possible to manipulate the combination of Cur and anthracyclines for a reduction in anthracycline toxicity and to overcome drug efflux via Pgp-mediated MDR in leukemia on AML leukemic cells and LSCs. Although Dox and Cur exhibit synergistic cytotoxic effects on cancer cell models, the combination of free Dox and free Cur has shown only a modest synergistic effect in vivo [37].

The aims of this study were determined the cytotoxicity of co-treatment with anthracycline drugs and curcumin for FLT3-overexpressing leukemic stem cells (KG-1a and KG1), FLT3-overexpressing leukemic cells (EoL-1), and non FLT3-expressing leukemic cells (U937). FLT3 protein is a member of the class III receptor tyrosine kinase (RTK) family [38]. It is overexpressed on the cell surface of AML leukemic stem cells and leukemic cells and plays an important role in cell survival and proliferation of leukemic cell blasts [39]. Cur has previously been shown to have an inhibitory effect on FLT3 protein expression in many types of FLT-3 expressing leukemic cell lines, such as EoL-1 and MV4-11 [32]. Thus, it was selected as a target protein for Dox–Cur treatment. Moreover, the effects of co-treatments on FLT3 protein expression and total cell numbers were determined.

## 2. Results

### 2.1. Determination of Cytotoxicity of Doxorubicin (Dox), Idarubicin (Ida), and Curcumin (Cur) on Leukemic Cell Viability by MTT Assay

**Figure 1:**
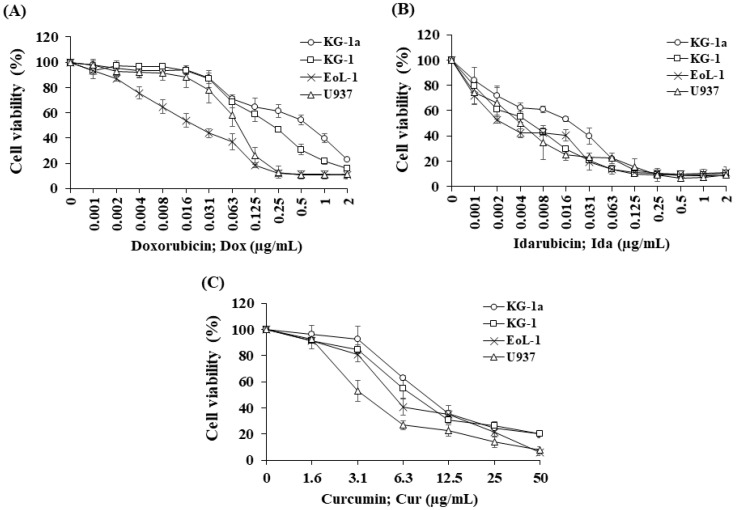
Cytotoxicity of (**A**) doxorubicin, (**B**) idarubicin, and (**C**) curcumin on KG-1a, KG-1, EoL-1, and U937 cells. The data are shown as mean ± SD from 3-time independent experiments.

A cell viability curve demonstrated Dox (Figure 1A) and IDa (Figure 1B) exhibited the highest cytotoxicity for EoL-1 cells, followed by U937, KG-1, and KG-1a cells. The cytotoxicity of all the treatments was assessed using an inhibitory concentration at a 50% growth (IC_50_) value. Ida demonstrated the greatest cytotoxic effects on KG-1a, KG-1, EoL-1, and U937 cells with IC_50_ values of 19.82 ± 1.80, 5.45 ± 0.89, 2.57 ± 0.32, and 4.73 ± 2.38 ng/mL, followed by Dox with IC_50_ values of 0.69 ± 0.12, 0.21 ± 0.02, 0.02 ± 0.01, and 0.08 ± 0.02 μg/mL, respectively. The IC_50_ values of all the chemotherapeutic drugs for the leukemic cell line models are shown in Table 1.

Cur was chosen to study the combination effect in order to improve the efficacy of Dox and Ida in AML treatment. For single treatment, Cur exhibited the highest cytotoxicity for U937cells, followed by EoL-1, KG-1, and KG-1a cells (Figure 1C). The IC_50_ values of Cur for KG-1a, KG-1, EoL-1, and U937 cells were 9.19 ± 0.49, 7.31 ± 1.45, 5.55 ± 0.46, and 3.55 ± 0.54 μg/mL, respectively (Table 1).

The IC_50_ values of Dox in leukemic stem cells (KG-1a and KG-1) were found to be significantly higher than for leukemic cells, EoL-1, and U937 cells. However, KG-1 leukemic stem cells were substantially more responsive to Dox and Ida than KG-1a cells, indicating a high number of LSCs affected the chemotherapeutic treatment’s sensitivity. Furthermore, the IC_50_ values of Cur in KG-1a cells were considerably higher than those in the other cells. These findings demonstrated the drug resistance in LSCs compared with LCs and suggested a possible route to improve the potency of traditional AML chemotherapeutics.

### 2.2. Determination of Cytotoxicity of Combined Doxorubicin–Curcumin (Dox–Cur) and Idarubicin–Curcumin (Ida–Cur) on Leukemic cell Viability by MTT Assay

Various doses of Dox and Ida (ranging from 0 to IC_80_ values) were added to Cur to investigate combination effects on the viability of AML cell lines. Dox was used to treat KG-1a (0–1.30 μg/mL), KG-1 (0–0.84 μg/mL), EoL-1 (0–0.08 μg/mL), and U937 (0–0.16 μg/mL) cells, and Ida was used to treat KG-1a (0–40.0 ng/mL), KG-1 (0–20.0 ng/mL), EoL-1 (0–8.0 ng/mL), and U937 (0–9.2 ng/mL) cells; these cells were cotreated with Cur at concentrations of the IC_20_ (condition 1), IC_30_ (condition 2), IC_40_ (condition 3), and IC_50_ (condition 4), respectively. The co-treatments of Dox–Cur and Ida–Cur exhibited higher cytotoxicity for KG-1a, KG-1, EoL-1, and U937 cells than single-drug treatments in a dose-dependent manner (see supplementary Figures S1 and S2). The IC_50_ value of each co-treatment demonstrated that curcumin enhanced the efficacy of doxorubicin and idarubicin in leukemic stem cells and non-leukemic stem cells, and also decreased the doses of drugs in co-treatment when compared with a single treatment. The IC_50_ values of Dox–Cur and Ida–Cur at different conditions in each cell line are shown in the supplementary Tables S1–S4.

### 2.3. Synergistic Effects of Combination Treatment

Using a combination index (CI) calculation, formulations 3 and 4 of Dox–Cur showed a synergistic effect (CI < 1) on KG-1a and EoL-1 cells and an additive effect (CI = 1) on U937 cells at the IC_50_ values, while most of the Ida–Cur treatments exhibited an antagonist effect (CI > 1) in all the leukemic cell lines (Table 2). It seemed Cur could not achieve high cytotoxicity in the Ida–Cur combination since Ida had an extremely low effective dose. Thus, most of the Ida–Cur-treated samples exhibited an antagonistic effect in AML leukemic cell lines.

### 2.4. Effects of Various Conditions of Combined Treatment of Dox–Cur at Concentration Value of IC_20_ on Cell Number and Cell Viability in FLT-3 Protein Expressing Leukemic Cells

Due to the synergistic and additive effect of Dox–Cur, a broader range of co-treatment conditions were chosen to investigate the effects on cell number and viability of FLT-3 protein-expressing AML leukemic cells, including KG-1a, KG-1 and EoL-1 cells. The IC_20_ values of the Dox treatments from the Section 2.2 were used to cotreat for 48 h with Cur. Dox–Cur conditions 1, 2, 3, and 4 (Dox (ng/mL) + Cur (μg/mL)) of KG-1a cells were 15.0 + 4.5, 16.0 + 5.5, 12.0 + 7.0, and 8.0 + 9.0, respectively. The conditions for KG-1 cells were 22.0 + 3.5, 10.0 + 4.5, 7.0 + 6.0, and 6.0 + 7.5, while the conditions of EoL-1 cells were 3.0 + 3.0, 0.7 + 4.0, 0.5 + 4.5, and 0.4 + 5.5, respectively. The results show that Dox concentrations at IC_20_ values and all co-treatment conditions consistently reduced the cell number of all leukemic cell lines (see supplementary Figures S3A–S5A). The total cell number of KG-1a cells (control group) was 2.59 × 10^5^ cells/mL, and cell number gradually decreased to 1.85 × 10^5^, 1.59 × 10^5^, 1.42 × 10^5^, 1.24 × 10^5^, and 0.98 × 10^5^ cells/mL in response to Dox and Dox–Cur treatment conditions 1, 2, 3, and 4, respectively. The total cell number of KG-1 cells decreased from 3.39 × 10^5^ cells/mL (control group) to 2.58 × 10^5^, 2.18 × 10^5^, 2.00 × 10^5^, 1.70 × 10^5^, and 1.44 × 10^5^ cells/mL in Dox and Dox–Cur treatment conditions 1, 2, 3, and 4, respectively. According to the cell number of EoL-1 cells after Dox and Dox–Cur conditions 1, 2, 3, and 4, the treatments reduced to 6.62 × 10^5^, 5.77 × 10^5^, 4.69 × 10^5^, 4.01 × 10^5^, and 3.27 × 10^5^ cells/mL, respectively, compared with 12.07 × 10^5^ cells/mL (control group). Cell viability of each sample was higher than 80% of the total cell count (see supplementary Figures S3B–S5B).

The total cell number of Cur treatments at the IC_20_, IC_30_, IC_40_, and IC_50_ values were observed to gradually decrease in all the cell lines in a dose dependent manner (see supplementary Figures S3C–S5C). Moreover, these data also corresponded to the decline in the cell numbers during the Dox–Cur treatment. The cell viability for each concentration of Cur was also higher than 80% of the total cell count (see supplementary Figures S3D–S5D).

### 2.5. Effects of Combined Treatments of Dox–Cur at Concentration Value of IC_20_ on FLT3 Protein Expressions in FLT-3 Protein Expressing Leukemic Stem Cells and Leukemic Cells

FLT3 protein is a member of the class III receptor tyrosine kinase (RTK) family [38]. It is overexpressed on the cell surface of AML leukemic stem cells and leukemic cells and plays an important role in cell survival and proliferation of leukemic cell blasts [39]. Cur has previously been shown to have an inhibitory effect on FLT3 protein expression in many types of FLT-3 expressing leukemic cell lines, such as EoL-1 and MV4-11 [32]. Thus, it was selected as a target protein for Dox–Cur treatment.

**Figure 2:**
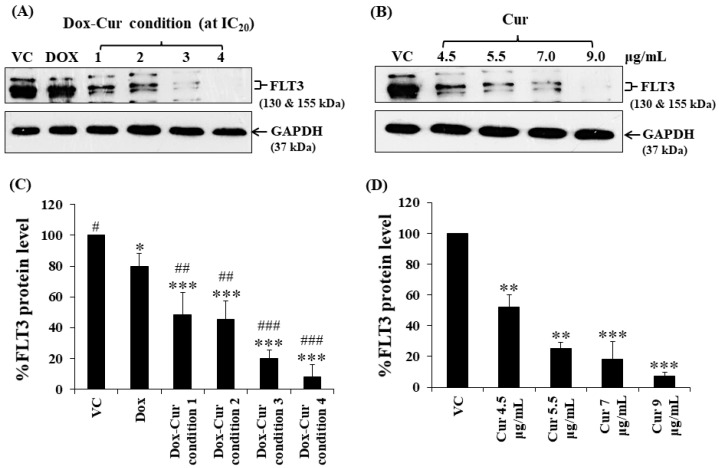
FLT3 protein expression of KG-1a cells after treatment with Dox and combined treatment of Dox–Cur at concentration value of IC_20_ for 48 h. Protein bands (**A**) and percentage (**C**) of FLT3 protein expression level of KG-1a cells treated with DMSO (VC), Dox at concentration of 60 ng/mL, Dox–Cur condition 1 (15 ng/mL Dox + 4.5 µg/mL Cur), Dox–Cur condition 2 (16 ng/mL Dox + 5.5 µg/mL Cur), Dox–Cur condition 3 (12 ng/mL Dox + 7 µg/mL Cur), and Dox–Cur condition 4 (8 ng/mL Dox + 9 µg/mL Cur) for 48 h. Whole protein lysates (80 μg/lane) were loaded onto SDS-PAGE. Protein bands (**B**) and percentage (**D**) of FLT3 protein expression level of KG-1a cells treated with DMSO (VC), Cur (4.5 µg/mL), Cur (5.5 µg/mL), Cur (7 µg/mL), and Cur (9 µg/mL) for 48 h. The data are shown as mean ± SD from 3 independent experiments. The significance of mean differences was assessed using one-way ANOVA. * *p* < 0.05, ** *p* < 0.01, and *** *p* < 0.001 compared with VC. ^#^ *p* < 0.05, ^##^ *p* < 0.01, and ^###^ *p* < 0.001 compared with single-Dox treatment.

**Figure 3:**
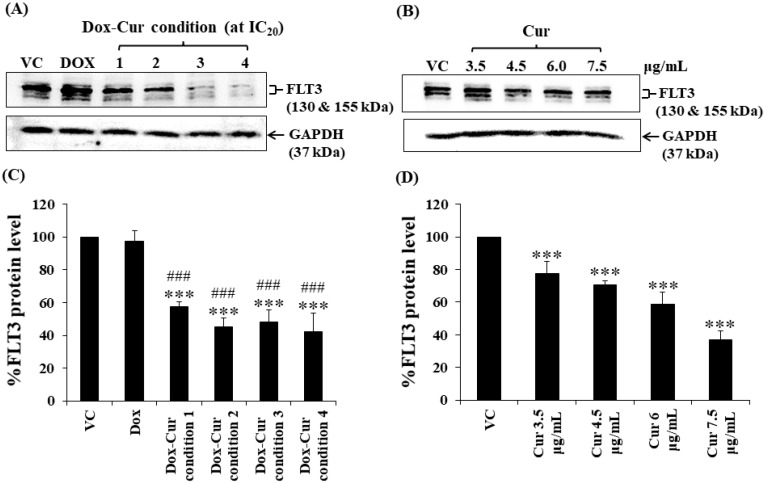
FLT3 protein levels of KG-1 cells after treatment with Dox and Dox–Cur at concentration value of IC_20_ for 48 h. Protein bands (**A**) and percentage (**C**) of FLT3 protein levels of KG-1 cells were from DMSO (VC), Dox (58 ng/mL), Dox–Cur condition 1 (22 ng/mL Dox + 3.5 µg/mL Cur), condition 2 (10 ng/mL Dox + 4.5 µg/mL Cur), condition 3 (7 ng/mL Dox + 6 µg/mL Cur), and condition 4 (6 ng/mL Dox + 7.5 µg/mL Cur). Protein bands (**B**) and percentage (**D**) of FLT3 protein level of KG-1 cells were from DMSO (VC), Cur (3.5 µg/mL), Cur (4.5 µg/mL), Cur (6 µg/mL), and Cur (7.5 µg/mL) for 48 h. Whole protein lysates (80 μg/lane) were loaded onto SDS-PAGE. The data are shown as mean ± SD from 3 independent experiments. The significance of mean differences was assessed using one-way ANOVA. *** *p* < 0.001 compared with VC. ^###^ *p* < 0.001 compared with single-Dox treatment.

**Figure 4:**
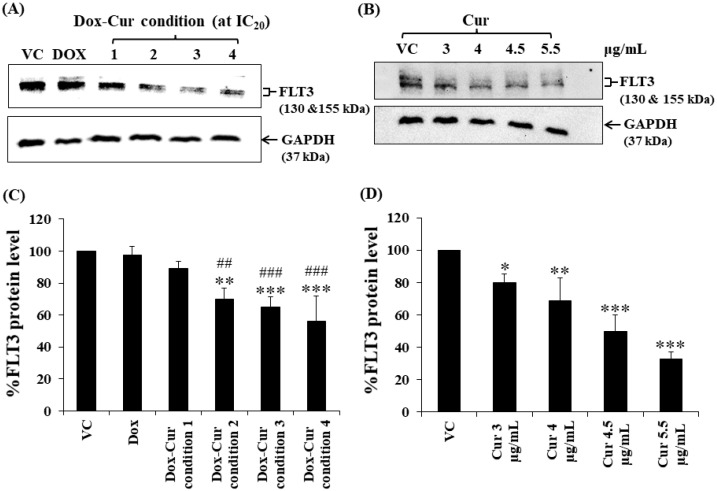
FLT3 protein expression of EoL-1 cells after treatment with Dox and combined treatment of Dox–Cur at concentration value of IC_20_ for 48 h. Protein bands (**A**) and percentage (**C**) of FLT3 protein expression level of EoL-1 cells treated with DMSO (VC), Dox at concentration of 6 ng/mL, Dox–Cur condition 1 (3 ng/mL Dox + 3 µg/mL Cur), Dox–Cur condition 2 (0.7 ng/mL Dox + 4 µg/mL Cur), Dox–Cur condition 3 (0.5 ng/mL Dox + 4.5 µg/mL Cur), and Dox–Cur condition 4 (0.4 ng/mL Dox + 5.5 µg/mL Cur) for 48 h. Whole protein lysates (80 μg/lane) were loaded onto SDS-PAGE. Protein bands (**B**) and percentage (**D**) of FLT3 protein expression level of EoL-1 cells treated with DMSO (VC), Cur (3 µg/mL), Cur (4 µg/mL), Cur (4.5 µg/mL), and Cur (5.5 µg/mL) for 48 h. The data are shown as Mean ± SD from 3 independent experiments. The significance of mean differences was assessed using one-way ANOVA. * *p* < 0.05, ** *p* < 0.01, and *** *p* < 0.001 compared with VC. ^##^ *p* < 0.01 and ^###^ *p* < 0.001 compared with single-Dox treatment.

In this study, KG-1a, KG-1, and EoL-1 cells were treated with Dox. Co-treatment was conditions at the IC_20_ value, and FLT3 protein expression levels were detected by Western blot. Dox and Dox–Cur co-treatment could decrease FLT-3 protein expression. In KG-1a cells, the FLT3 protein levels of Dox and Dox–Cur treatment conditions 1, 2, 3, and 4 were decreased by 20.4 ± 8.8%, 51.6 ± 14.5%, 54.6 ± 12.2%, 80.2 ± 5.7%, and 92.2 ± 8.2%, respectively (Figure 2A,C). For KG-1 cells, FLT3 proteins were gradually decreased by 2.7 ± 6.5%, 42.3 ± 2.9%, 55.0 ± 5.4%, 52.0 ± 7.3%, and 57.9 ± 11.5% in respond to Dox and Dox–Cur conditions 1, 2, 3, and 4, respectively (Figure 3A,C). Similarly, FLT3 protein expression in EoL-1 cells was reduced to 2.7 ± 5.6%, 10.7 ± 4.2%, 29.9 ± 6.8%, 35.2 ± 6.4%, and 43.7 ± 15.7% in response to Dox and Dox–Cur conditions 1, 2, 3, and 4, respectively, compared with the control group (100% expression level; Figure 4A,C). Additionally, the ability of each concentration of Cur in the combination treatment to suppress FLT3 protein expression was evaluated. All concentrations of Cur treatments were also able to decrease the protein expression levels compared with the control group in a dose-dependent manner (Figure 2B,D, Figure 3B,D and Figure 4B,D).

### 2.6. Effects of Combination Treatments of Various Concentrations of Cur and a Fixed Concentration of Dox on Cell Number and Viability in Leukemic Stem Cells and Leukemic Cells

**Figure 5:**
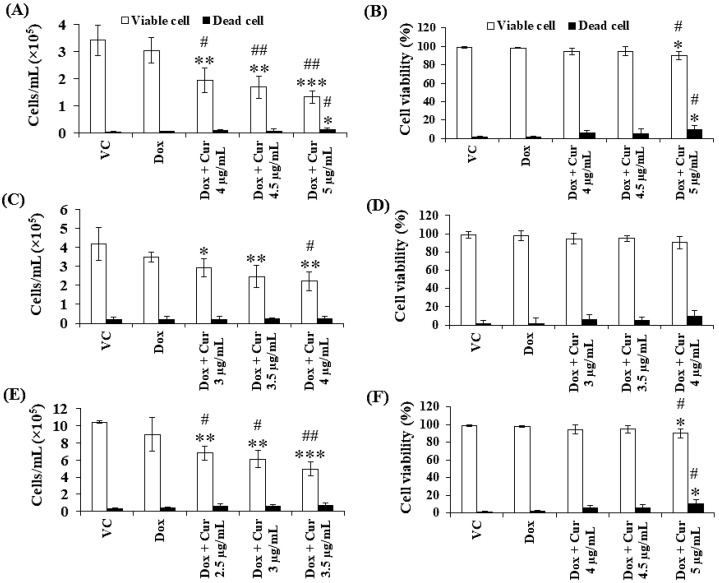
Total cell number and cell viability of KG-1a, KG-1, and EoL-1 cells after treatment with Dox and combination of fixed concentration of Dox and various non-toxic concentration of Cur for 48 h. Total cell number (**A**) and cell viability (**B**) of KG-1a cells were treated with DMSO (VC), Dox (15 ng/mL), Dox + Cur (4 µg/mL), Dox + Cur (4.5 µg/mL), and Dox + Cur (5 µg/mL) for 48 h. Total cell number (**C**) and cell viability (**D**) of KG-1 cells were treated with DMSO (VC), Dox (22 ng/mL), Dox and Cur (3 µg/mL), Dox and Cur (3.5 µg/mL), and Dox and Cur (4 µg/mL) for 48 h. Total cell number (**E**) and cell viability (**F**) of EoL-1 cells were treated with DMSO (VC), Dox (2.8 ng/mL), Dox and Cur (2.5 µg/mL), Dox and Cur (3 µg/mL), and Dox and Cur (3.5 µg/mL) for 48 h. The data are shown as mean ± SD from 3 independent experiments. The significance of mean differences was assessed using one-way ANOVA. * *p* < 0.05, ** *p* < 0.01, and *** *p* < 0.001 compared with VC. ^#^ *p* < 0.05 and ^##^ *p* < 0.01 compared with single-Dox treatment.

Thus far, Cur and Dox–Cur treatments were found to inhibit AML LSC and LC cell proliferation more effectively than Dox treatment alone. Thus, three non-toxic concentrations within the range of Cur IC_20_ value and a fixed concentration of Dox from Dox–Cur condition 1 were tested using KG-1a, KG-1, and EoL-1 cells for 48 h. Co-treatments of Dox–Cur significantly decreased the cell number of both cell lines in a dose-dependent manner when compared with a single-Dox treatment and control (Figure 5). The cell number of KG-1a cells in the control group was 3.44 × 10^5^ cells/mL, and decreased to 2.96 × 10^5^, 2.21 × 10^5^, 1.91 × 10^5^, and 1.46 × 10^5^ cells/mL in response to Dox, Dox + Cur at 4 µg/mL, Dox + Cur at 4.5 µg/mL, and Dox + Cur at 5 µg/mL, respectively (Figure 5A). In addition, the cell number of KG-1 cells decreased from 4.18 × 10^5^ cells/mL in the control group to 3.48 × 10^5^, 2.93 × 10^5^, 2.47 × 10^5^, and 2.22 × 10^5^ cells/mL in response to Dox, Dox + Cur at 3 µg/mL, Dox + Cur at 3.5 µg/mL, and Dox + Cur at 4 µg/mL, respectively (Figure 5C). Moreover, the number of EoL-1 cells also decreased from 10.43 × 10^5^ cells/mL in the control group to 8.99 × 10^5^, 6.82 × 10^5^, 6.08 × 10^5^, and 4.94 × 10^5^ cells/mL in response to the treatments of Dox, Dox + Cur at 2.5 µg/mL, Dox + Cur at 3 µg/mL, and Dox + Cur at 3.5 µg/mL, respectively (Figure 5E). All samples exhibited viable cells higher than 80% of the total cell count (Figure 5B,D,F).

### 2.7. Effects of Combination Treatments of Various Concentrations of Cur and a Fixed Concentration of Dox on FLT3 Protein Expressions in Leukemic Stem Cells

**Figure 6:**
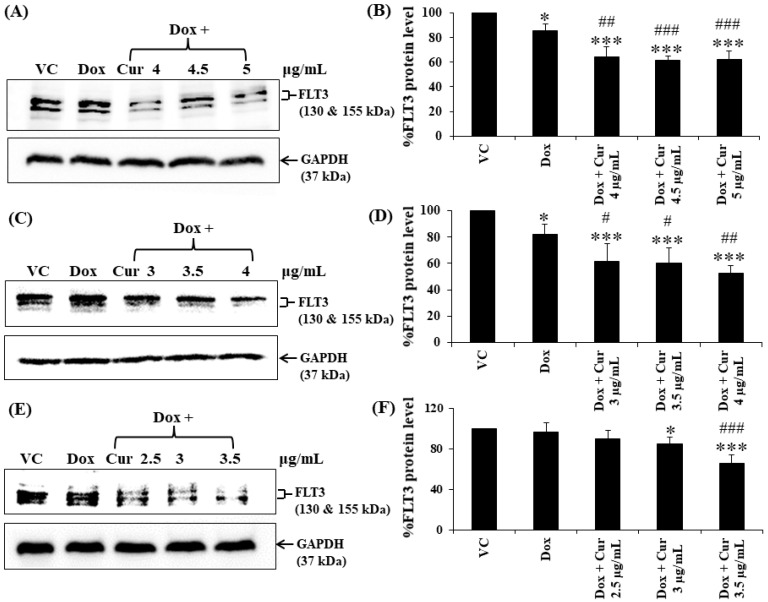
FLT3 protein expression of KG-1a, KG-1, and EoL-1 cells following treatment with Dox and combination of fixed concentration of Dox and various non-toxic concentrations of Cur for 48 h. Protein bands (**A**) and percentage (**B**) of KG-1a cells treated with DMSO (VC), Dox (15 ng/mL), Dox + Cur (4 µg/mL), Dox + Cur (4.5 µg/mL), and Dox + Cur (5 µg/mL), for 48 h. Protein band (**C**) and percentage (**D**) of KG-1 cells treated with DMSO (VC), Dox (22 ng/mL), Dox + Cur (3 µg/mL), Dox + Cur (3.5 µg/mL), and Dox + Cur (4 µg/mL) for 48 h. Protein band (**E**) and percentage (**F**) of EoL-1 cells were treated with DMSO (VC), Dox (2.8 ng/mL), Dox + Cur (2.5 µg/mL), Dox + Cur (3 µg/mL), and Dox + Cur (3.5 µg/mL) for 48 h. Whole protein lysates (80 μg/lane) were loaded onto SDS-PAGE. The data are shown as mean ± SD from 3 independent experiments. The significance of mean differences was assessed using one-way ANOVA. * *p* < 0.05 and *** *p* < 0.001 compared with VC. ^#^ *p* < 0.05, ^##^ *p* < 0.01, and ^###^ *p* < 0.001 compared with single-Dox treatment.

Cur and Dox–Cur co-treatments were more effective in suppressing FLT3 protein expression in all AML leukemic cell lines than single-Dox treatment. Moreover, the percentages of FLT3 protein in all conditions of co-treatments were also similar to those of Cur treatment alone. To confirm the effect of Cur in increasing the inhibitory effect of Dox on FLT3 expression, co-treatment of Dox–Cur condition 1 was used. Western blot clearly showed all the non-toxic concentrations of Cur remarkably increased FLT3 protein expression in all three AML cell lines when combined with Dox. The FLT3 expression level of KG-1a cells after treatment with Dox (15 ng/mL), Dox + Cur (4 µg/mL), Dox + Cur (4.5 µg/mL), and Dox + Cur (5 µg/mL) were decreased by 14.1 ± 5.2%, 35.8 ± 8.5%, 38.2 ± 3.3%, and 37.8 ± 7.0%, respectively, compared with the vehicle control (100% FLT3 protein expression level) (Figure 6A,B). For KG-1 cells, the FLT3 protein levels were decreased by 17.9 ± 7.6%, 38.2 ± 13.0%, 39.7 ± 11.4%, and 47.2 ± 5.4% in response to Dox (22 ng/mL), Dox + Cur (3 µg/mL), Dox + Cur (3.5 µg/mL), and Dox + Cur (4 µg/mL), respectively (Figure 6C,D). Finally, while the FLT3 protein expression levels in EoL-1 cells were reduced to 3.5 ± 8.9%, 10.2 ± 8.1%, 15.6 ± 7.1%, and 34.6 ± 8.9% in response to the treatments of Dox (2.8 ng/mL), Dox + Cur (2.5 µg/mL), Dox + Cur (3 µg/mL), and Dox + Cur (3.5 µg/mL), respectively, from 100% protein expression level of the vehicle control (Figure 6E,F).

## 3. Discussion

Doxorubicin (Dox) and idarubicin (Ida) are the standard chemotherapy treatments for AML patients. These compounds can destroy leukemic cells by binding to DNA and inhibiting topoisomerase II activity in DNA transcription, thereby triggering apoptosis or autophagy [16,40,41]. The cytotoxic activity of these anthracyclines was determined in each leukemic cell line by MTT assays. Both drugs showed the greatest cytotoxicity for EoL-1 cells, followed by U937, KG-1, and KG-1a cells. The inhibitory concentrations at cell growth values of 50 (IC_50_) for Ida on KG-1a and KG-1 cells were 19.82 ± 1.80 and 5.45 ± 0.89 ng/mL, respectively. In contrast, Dox showed lower cytotoxicity than Ida with IC_50_ values of 0.65 ± 0.13 and 0.21 ± 0.02 µg/mL, respectively. Ida and Dox doses in a previous report in vitro were 1–100 ng/mL and 0.1–1.5 μg/mL, respectively, in normal and leukemic human bone marrow progenitors [42]. Drug doses were within the range of our studies. However, doses used in this study presented activity at low levels when compared with the previous report. Looking at intravenous injections of Ida and Dox in a rat model, the maximum tolerated doses of both drugs were 3 mg/kg and 0.75 mg/kg per injection. Moreover, the cardiac toxicity of Ida remained significantly lower than that of Dox [43].

Next, studies were conducted to determine the effect of combining the natural product curcumin with these AML chemotherapeutics. Cur tended to increase the cytotoxicity of Dox and Ida on all leukemic cell lines; however, several conditions of Dox + Cur co-treatment showed additive and synergistic manners, whereas most conditions of Ida + Cur co-treatment suggested that Cur was antagonistic. Ida appears to have a mechanism similar to Dox in that it can intercalate DNA and block topoisomerase II activity [44], thus differences may be due to the chemical structures of Ida and Dox. The absence of the methoxyl group at position 4 of idarubicin’s structure increased the lipophilicity and rate of cellular uptake, leading to greater toxicity than that of daunorubicin or doxorubicin [45]. Furthermore, Ida is relatively more potent than doxorubicin in suppressing the growth of low or nonproliferating progenitor cells [42]. Thus, it is likely that the concentrations of Cur used in the combination treatment were too low to achieve a synergistic effect when combined with Ida. As a result, increasing the concentration of Cur combined with Ida may be explored in future research.

In addition, the cytotoxicity of curcumin (Cur), a natural substance with chemosensitizing and chemoprotective activities [26], was also examined with four leukemic cell lines by MTT assay. Cur demonstrated the highest cytotoxic effect on U937 cells, followed by EoL-1, KG-1, and KG-1a cells. Thus, Cur was selected as a supplementary substance for enhancing the efficiency and decreasing the toxicity of anthracycline drugs in this study.

Appropriate concentrations of Dox, Ida, and Cur were chosen for the combination effect, based on the results of the preliminary screening study. The co-treatments of Ida and Dox at concentrations in the range of IC_10_ to IC_50_ values and low concentrations of Cur at 1, 2, and 3 μg/mL did not show a different effect on cell viability of KG-1a and KG-1 cells, when compared with a single treatment (data not shown). Thus, KG-1a, KG1-a, EoL-1, and U937 cells were treated with Dox and Ida at the concentration values of IC_10_–IC_80_ combined with Cur at concentrations of IC_20_, IC_30_, IC_40_, and IC_50_ values, respectively. To investigate the combination effect, the percentage of cell viability of each treatment was calculated and compared with that for the single drug and vehicle control, and the inhibitory concentrations at 20% (IC_20_) and 50% (IC_50_) were determined.

Co-treatment of Dox–Cur and Ida–Cur tended to increase the cytotoxicity for KG-1a, KG-1, EoL-1, and U937 cells in dose-dependent manners as compared with single-drug treatment. Moreover, Cur also enhanced the cytotoxic efficacy for both chemotherapeutic drugs in dose dependent manners, based on the lower IC_50_ values of anthracyclines used in co-treatment in each cell line.

Dox and Ida are usually ineffective due to an increase in LSCs, drug resistance, and relapse in AML patients. In this study, the natural substance Cur was found to improve the cytotoxicity of Dox and Ida in all the cell lines due to its anti-leukemic (apoptotic induction) [46] and chemosensitizing (decreasing MDR-1 gene expression) [34] activities. For these reasons, Cur improved both chemotherapies by lowering IC_50_ values for Dox and Ida in co-treatments when compared with single drug treatments.

It is notable that effective doses of the co-treatments used to treat KG-1a cells were higher than those for KG-1, EoL-1, and U937 cells. KG-1a and KG-1 cells are leukemic stem cell lines with a high percentage of leukemic stem cells (~95% and ~55%, respectively). These cells are well-known for their chemotherapy resistance, which includes a prolonged stage G_0_ of the cell cycle and high expression of the drug efflux pump. Since the EoL-1 and U937 cells lack these stem cell features, they were more vulnerable to the co-treatments.

The combination treatment of Dox–Cur showed synergistic and additive cytotoxic effects on both AML leukemic stem cell lines (KG-1a and KG-1 cells) and AML leukemic cell lines (EoL-1 and U937 cells). Even though only Dox–Cur condition 3 showed synergism on KG-1a and EoL-1 cells, Cur was able to lower chemotherapeutic agent doses. The combination treatment also reduced the concentration at the IC_50_ value of Dox in each cell line which could be a useful formulation to decrease the cytotoxicity of Dox on normal cells. However, the poor solubility and short biological half-life of Cur, as well as the non-specific activity of Dox, may result in low absorption and cytotoxicity of these drugs in tumor cells [16,27].

FLT3 is a key driver of AML, and its mutations are associated with the development of high risk of relapse in patients. Previous studies demonstrated that Cur has an inhibitory effect on FLT3 protein expression in leukemic cells [32]. Thus, the combination of Dox and Cur for AML treatment may lead to FLT3 protein expression reduction, which in turn could denote reduced proliferation of leukemic cells.

In this study, non-toxic doses at IC_20_ of the four conditions of Dox–Cur co-treatments decreased the cell number and showed a higher inhibitory effect on FLT3 protein expression than single Dox in both stem cell and leukemic cell lines. However, when compared with Cur alone, none of the co-treatments showed any differences. To confirm, Dox–Cur condition 1 was selected for the inhibitory effect of various concentrations of Cur with a fixed concentration of Dox on cell number and FLT3 protein expression. Dox–Cur-treated samples exhibited a lower cell number and FLT3 protein level than treatment with Dox alone in all cell lines. It is possible that Cur was the main compound in co-treatment that could suppress the FLT3 protein expression in a dose-dependent manner, leading to a decrease in cell proliferation, while the main functions of Dox, in order to eradicate tumor cells, involved inhibiting cell cycle progression, producing oxidation, and inducing apoptosis, which is unrelated to the inhibition of FLT3 protein expression [16]. In addition, these functions of Dox could affect cell proliferation, resulting in the decrease in cell number in single-Dox-treated samples. The co-treatment had an inhibitory effect on FLT3 protein expression. Notably, the IC_20_ values of Dox in Dox–Cur condition 1 (Dox 15 ng/mL + Cur 4.5 µg/mL) and 2 (Dox 16 ng/mL + Cur 5.5 µg/mL) in KG-1a cells were not reduced in a dose-dependent response to the Cur increase. This might be because the chosen Cur concentration was too low, making the lowering of the Dox concentration in the reaction impractical, as shown by the cell number and FLT3 protein expression level.

Co-treatment likely assists in enhancing the cytotoxic effects of Dox by inhibiting the cell proliferation activity of AML leukemic stem cells and leukemic cells as a result of the decrease in the cell proliferation rate of each co-treatment condition compared with the single treatment and vehicle control. These findings are consistent with a previous study in which the combination of Dox with SU11657, a FLT3 inhibitor, increased the survival rate of APL mice and overcame resistance to traditional chemotherapies in AML [47].

## 4. Materials and Methods

### 4.1. Reagents and Chemicals

Curcumin (Cur) was purchased from Thai-China Flavours and Fragrances Industry Co., LTD (Nonthaburi, Thailand). MTT dye (3-[4,5-dimethylthiazol-2-yl]-2,5-diphenyl-tetrazolium bromide), trypan blue, and DMSO were purchased from Sigma-Aldrich (St. Louis, MO, USA). IMDM, RPMI-1640, penicillin-streptomycin, L-glutamine, and fetal calf serum were purchased from Invitrogen^TM^ Life (Carlsbad, CA, USA). Rabbit polyclonal anti-GAPDH was purchased from Santa Cruz Bitechnology (Santa Cruz, CA, USA). HRP-conjugated goat anti-rabbit IgG was purchased from Promega (Madison, WI, USA). Rabbit polyclonal anti-FLT3 and Luminata^TM^ Forte Western HRP Substrate were purchased from Merck Millipore Corporation (Billerica, MA, USA).

### 4.2. Cell Culture

KG-1a (acute myeloblastic leukemic cell line; ATCC^®^ CCL-246.1™), KG-1 (acute myeloblastic leukemic cell line; ATCC^®^ CCL-246™), and EoL-1 (acute myeloblastic leukemic cell line) were used as human leukemic cell line models in this study. KG-1a and KG-1 cells were cultured in IMDM medium (Invitrogen^TM^, CA, USA) supplemented with 20% fetal bovine serum, 2 mM L-glutamine, 100 units/mL penicillin, and 100 µg/mL streptomycin. EoL-1 (Eosinophilic leukemic cell line), a model of FLT3 overexpressing leukemic cells, was purchased from RIKEN BRC Cell Bank (Ibaraki, Japan). U937 (monoblastic leukemic cell line) was purchased from ATCC^®^. These were cultured in RPMI-1640 medium containing 10% fetal calf serum, 1 mM L-glutamine, 100 units/mL penicillin, and 100 μg/mL streptomycin. All the leukemic cell lines were cultured at 37 °C in a humidified incubator with 5% CO_2_.

### 4.3. Cytotoxicity of Single Doxorubicin, Idarubicin, and Curcumin (Curcuminoid Mixture) on Leukemic Stem Cell and Leukemic Cell Viability by MTT Assay

KG-1a and KG-1 cell lines were adjusted to 1.5 × 10^4^ cells, while EoL-1 and U937 cells were adjusted to 3.0 × 10^4^ and 1.0 × 10^4^ cells in 100 µL of complete medium, and then seeded into flat-bottom 96-well plate and incubated at 37 °C under 5% CO_2_ atmosphere for 24 h. Following that, doxorubicin (Dox), idarubicin (Ida), and curcumin (Cur) were diluted in 100 µL of medium with the 2-fold dilution technique and applied to the cells to obtain the final concentrations from 0.001 to 2 µg/mL for Dox and Ida and 1.56 to 50 µg/mL for Cur for 48 h. Complete medium and DMSO were used as cell control and vehicle control, respectively. Afterwards, 100 µL of medium was removed, and 15 µL of MTT dye solution was added and further incubated for 4 h. After removing the supernatant, 200 µL of DMSO were added to dissolve the formazan crystals (cell viability indication). The optical density was measured using an ELISA plate reader at 578 nm with the reference wavelength at 630 nm. The percentage of cell survival was calculated from the absorbance of test and control wells using the equation below, and the inhibitory concentration at 20% (IC_20_) and 50% (IC_50_) growth of Dox, Ida, and Cur were determined.

$$\%\;\mathrm{Cell}\;\mathrm{viability}=\frac{\mathrm{Absorbance}\;\mathrm{of}\;\mathrm{test}}{\mathrm{Absorbance}\;\mathrm{of}\;\mathrm{vehicle}\;\mathrm{control}}\times 100$$

### 4.4. Assessing Cytotoxic Effects of Combination of the Chemotherapeutic Drug and Curcumin on Leukemic Stem Cell and Leukemic Cell Viability by MTT Assay

KG-1a, KG-1, EoL-1, and U937 cells were seeded into flat-bottom 96-well plate and incubated at 37 °C under 5% CO_2_ atmosphere for 24 h. Then, various concentrations of Dox and Ida in the range of IC_10_–IC_80_ (from the cytotoxic effects of single treatment) were mixed with each concentration of Cur at IC_20_, IC_30_, IC_40_, and IC_50_ values, as well as DMSO, to prepare the combination and single drug treatments, respectively. All the treatments were added to the cells and incubated for 48 h. The cell viability in each treatment was determined by the MTT assay, as described in Section 4.3.

### 4.5. Synergistic Effects of Combination Treatment

The combination index (CI) is used to quantitatively define the synergistic (CI < 1), additive (CI = 1), and antagonist effect (CI > 1) of a drug–drug interaction [48]. It can be calculated by using the following equation:

$$\mathrm{CI}=\frac{\mathrm{Dose}\;\mathrm{of}\;\mathrm{drug}\;\mathrm{in}\;\mathrm{combination}\;\mathrm{at}\;\mathrm{ICx}}{\mathrm{Dose}\;\mathrm{of}\;\sin\mathrm{gle}\;\mathrm{drug}\;\mathrm{at}\;\mathrm{ICx}}\times \frac{\mathrm{Dose}\;\mathrm{of}\;\mathrm{Cur}\;\mathrm{in}\;\mathrm{combination}\;\mathrm{at}\;\mathrm{ICx}}{\mathrm{Dose}\;\mathrm{of}\;\sin\mathrm{gle}\;\mathrm{Cur}\;\mathrm{at}\;\mathrm{ICx}}$$

ICx = The concentrations required to produce the given effect, such as IC_50_.

### 4.6. Cell Number and Cell Viability of FLT3-Exprssing Cells Determined by the Trypan Blue Exclusion Method

KG-1a, KG-1, and EoL-1 cells were adjusted to 1.5 × 10^5^ and 3.0 × 10^5^ cells/mL, respectively, and incubated with non-toxic concentrations (IC_20_) of Dox, Cur, and combination treatment at 37 °C under 5% CO_2_ atmosphere for 48 h. Then, the treated cells were collected, and their cell number and percent of cell viability were estimated using the trypan blue exclusion method by mixing the cells and the 0.4% trypan blue solution in a ratio of 1:1; following this, the cells were counted in a hemacytometer under a light microscope.

### 4.7. Western Blotting

KG-1a, KG-1, and EoL-1 cells were prepared and treated with Dox, Cur, and the co-treatment as discussed in Section 4.6. After that, the cells were harvested after 48 h of incubation, and the whole proteins were extracted using RIPA buffer. The protein concentration was measured with the Folin-Lowry method. The protein lysates were separated through 7.5% SDS-PAGE and then transferred to PVDF membranes. For the antibody–protein reaction step, the membrane was cut to separate FLT3 (target protein) and GAPDH (internal control protein), and then blocked in 5% skim milk. The part of the membrane containing FLT3 protein was probed with rabbit polyclonal anti-FLT3 at a dilution 1:1000, whereas the part containing GAPDH was probed with rabbit polyclonal anti-GAPDH antibody at a dilution of 1:1000. After that, the reaction was followed by a 1:15,000 dilution of HRP-conjugated goat anti-rabbit IgG. The proteins were visualized using Luminata™ Forte Western HRP substrate. Finally, the protein band signal (chemiluminescence) was detected by X-ray film or Fluorchem E Western blot and gel imager (ProteinSimple, San Jose, CA, USA) and quantified using a scan densitometer (Bio-Rad, Hercules, CA, USA) or Fluorchem Q program (ProteinSimple, CA, USA).

### 4.8. Statistical Analysis

The average of triplicate experiments and standard derivation (SD) were used for quantification. The levels of target protein expressions were compared with those of the vehicle control in each experiment. The results are shown as mean ± SD. The differences between the means of each sample were analyzed by one-way analysis of variance (one-way ANOVA). Statistical significance was considered at *p* < 0.05, *p* < 0.01, and *p* < 0.001.

## 5. Conclusions

Overall, anthracyclines (Dox and Ida) and Cur, a natural phenolic compound with anti-tumor activity, were shown to be effective AML chemotherapeutic agents. The combination of Dox and Cur had a synergistic effect and could improve Dox anti-tumor activity in AML cells, particularly leukemic stem cells, by inhibiting cell proliferation through FLT-3 protein suppression. This study demonstrated the benefit of co-treatment combining the natural product curcumin and chemotherapeutic drugs (Dox and Ida) in leukemia therapy as a potential approach to decrease chemotherapy dose and thereby reduce associated side effects. Adding nontoxic doses of edible Cur to chemotherapeutic drugs enhanced the cytotoxicity of Dox and suppressed leukemic stem cell proliferation. This finding presents an alternative choice that may be useful in the development of a promising regimen for the treatment of AML relapse in the future.

## Figures and Tables

**Table 1:** The IC_50_ values of chemotherapeutic drugs and curcumin on KG-1a, KG-1, EoL-1, and U937 cells.

Treatment	IC_50_ Value			
	KG-1a	KG-1	EoL-1	U937
Idarubicin (Ida) (ng/mL)	19.82 ± 1.80 ^###^	5.45 ± 0.89 ***	2.57 ± 0.32 ***	4.72 ± 2.38 ***
Doxorubicin (Dox) (μg/mL)	0.69 ± 0.12 ^###^	0.21 ± 0.02 ***	0.02 ± 0.01 ***^,##^	0.08 ± 0.02 ***^,#^
Curcumin (Cur) (μg/mL)	9.19 ± 0.49 ^#^	7.31 ± 1.45 *	5.55 ± 0.46 **^,#^	3.55 ± 0.54 ***^,##^

The significance of mean differences was assessed using one-way ANOVA. * *p* < 0.05, ** *p* < 0.01, and *** *p* < 0.001 compared with KG-1a cells. ^#^ *p* < 0.05, ^##^ *p* < 0.01, and ^###^ *p* < 0.001 compared with KG-1 cells.

**Table 2:** IC_50_ values of co-treatment of Dox–Cur and Ida–Cur on KG-1a, KG-1, EoL-1, and U937 cells.

Cell Line	Dox–Cur	CI Value	Ida–Cur	CI Value
KG-1a	Dox + Cur 1 (4.5 μg/mL)	1.08	Ida + Cur 1(4.5 μg/mL)	1.16
	Dox + Cur 2 (5.5 μg/mL)	1.12	Ida + Cur 2 (5.5 μg/mL)	1.27
	Dox + Cur 3 (7.0 μg/mL)	0.97	Ida + Cur 3 (7.0 μg/mL)	1.21
	Dox + Cur 4 (9.0 μg/mL)	1.02	Ida + Cur 4 (9.0 μg/mL)	1.09
KG-1	Dox + Cur 1 (3.5 μg/mL)	1.36	Ida + Cur 1 (3.5 μg/mL)	1.44
	Dox + Cur 2 (4.5 μg/mL)	1.07	Ida + Cur 2 (4.5 μg/mL)	1.55
	Dox + Cur 3 (6.0 μg/mL)	1.04	Ida + Cur 3 (6.0 μg/mL)	1.28
	Dox + Cur 4 (7.5 μg/mL)	1.07	Ida + Cur 4 (7.5 μg/mL)	1.11
EoL-1	Dox + Cur 1 (3.0 μg/mL)	1.23	Ida + Cur 1 (3.5 μg/mL)	1.27
	Dox + Cur 2 (4.0 μg/mL)	1.12	Ida + Cur 2 (4.0 μg/mL)	1.24
	Dox + Cur 3 (4.5 μg/mL)	0.92	Ida + Cur 3 (4.5 μg/mL)	0.85
	Dox + Cur 4 (5.5 μg/mL)	1.03	Ida + Cur 4 (5.5 μg/mL)	1.03
U937	Dox + Cur 1 (2.0 μg/mL)	1.46	Ida + Cur 1 (2.0 μg/mL)	1.35
	Dox + Cur 2 (2.5 μg/mL)	1.55	Ida + Cur 2 (2.5 μg/mL)	1.40
	Dox + Cur 3 (3.0 μg/mL)	1.42	Ida + Cur 3 (3.0 μg/mL)	1.26
	Dox + Cur 4 (3.5 μg/mL)	1.00	Ida + Cur 4 (3.5 μg/mL)	1.04

## Data Availability

Not applicable.

## Supplementary Materials

The following are available online, Figure S1: Cytotoxic effects of co-treatment of doxorubicin and curcumin (Dox–Cur) on KG-1a, KG-1, EoL-1, and U937 cell lines, Figure S2: Cytotoxic effects of co-treatment of Idarubicin and curcumin (Ida–Cur) on KG-1a, KG-1, EoL-1, and U937 cell lines, Figure S3: Cell number and cell viability of KG-1a cells after treatment with Dox, Cur, and combined treatment of Dox–Cur for 48 h, Figure S4: Cell number and cell viability of KG-1 cells after treatment with Dox, Cur, and combined treatment of Dox–Cur for 48 h, Figure S5: Cell number and cell viability of EoL-1 cells after treatment with Dox, Cur, and combined treatment of Dox–Cur for 48 h, Table S1: IC_50_ values of single and co-treatment of Dox or Ida and Cur on KG-1a cells, Table S2: IC_50_ values of single and co-treatment of Dox or Ida and Cur on KG-1 cells, Table S3: IC_50_ values of single and co-treatment of Dox or Ida and Cur on EoL-1 cells, Table S4: IC_50_ values of single and co-treatment of Dox or Ida and Cur on U937 cells.

## Sample Availability

Not available.
