# Supplementary material for: Co-Treatments of Edible Curcumin from Turmeric Rhizomes and Chemotherapeutic Drugs on Cytotoxicity and FLT3 Protein Expression in Leukemic Stem Cells
Source: Molecules. 2021 Sep 24;26(19):5785. doi: 10.3390/molecules26195785 (PMC8510311; doi:10.3390/molecules26195785)
Supplement: Supplementary file 1 [file molecules-26-05785-s001.zip › molecules-1370288-supplementary.pdf]

Supplementary data

Co-treatments of Edible Curcumin from Turmeric Rhizomes and Chemotherapeutic Drugs on Cytotoxicity and FLT3 Protein Expression in Leukemic Stem Cells

Fah Chueahongthong [1,†], Singkome Tima [1,2,3,†], Sawitree Chiampanichayakul [1,2], Cory Berkland [4,*] and Songyot Anuchapreeda [1,2,3,*]

[1] Division of Clinical Microscopy, Department of Medical Technology, Faculty of Associated Medical Sciences, Chiang Mai University, Chiang Mai 50200, Thailand; fahmyfah@hotmail.com (F.C.); singkome@gmail.com (S.T.); chiampanich@gmail.com (S.C.)
[2] Cancer Research Unit of Associated Medical Sciences (AMS-CRU), Faculty of Associated Medical Sciences, Chiang Mai University, Chiang Mai 50200, Thailand; singkome@gmail.com (S.T.); chiampanich@gmail.com (S.C.)
[3] Center for Research and Development of Natural Products for Health, Chiang Mai University, Chiang Mai 50200, Thailand; singkome@gmail.com (S.T.)
[4] Department of Pharmaceutical Chemistry, School of Pharmacy, University of Kansas, Kansas 66047 United States; berkland@ku.edu (C.B.)
† The first two authors (F.C. and S.T.) contributed as co-first author.

Abstract: This study aims to enhance efficacy and reduce toxicity of the combination treatment of a drug and curcumin (Cur) on leukemic stem cell and leukemic cell lines, including KG-1a and KG-1 ($FLT3^+$ LSCs), EoL-1 ($FLT3^+$ LCs), and U937 ($FLT3^-$ LCs). The cytotoxicity of co-treatments of doxorubicin (Dox) or idarubicin (Ida) at concentrations of the IC_{10} – IC_{80} values and each concentration of Cur at the IC_{20}, IC_{30}, IC_{40}, and IC_{50} values (conditions 1, 2, 3, and 4) was determined by MTT assays. Dox–Cur and Ida-Cur increased cytotoxicity in leukemic cells. Dox–Cur co-treatment showed additive and synergistic effects in several conditions. The effect of this co-treatment on FLT3 expression in KG-1a, KG-1, and EoL-1 cells was examined by Western blotting. Dox–Cur decreased FLT3 protein levels and total cell numbers in all the cell lines in a dose-dependent manner. In summary, this study exhibits a novel report of Dox-Cur co-treatment in both enhancing cytotoxicity of Dox and inhibiting cell proliferation via FLT3 protein expression in leukemia stem cells and leukemic cells. This is the option of leukemia treatment with reducing side effects of chemotherapeutic drugs to leukemia patients.

Keywords: leukemia; leukemic stem cell; FLT-3; chemotherapeutic drug; curcumin; co-treatment

Correspondance to:

Prof. Dr. Songyot Anuchapreeda
Division of Clinical Microscopy, Department of Medical Technology, Faculty of Associated Medical Sciences, Chiang Mai University, Chiang Mai, 50200, Thailand
Tel: (66) 53-949237
Fax: (66) 53-936042
E-mail: sanuchapreeda@gmail.com

Prof. Dr. Cory Berkland
Department of Pharmaceutical Chemistry, School of Pharmacy, University of Kansas, Kansas 66047, US.
Tel.: +785 864 1455
Fax: +785 864 1454
E-mail address: berkland@ku.edu

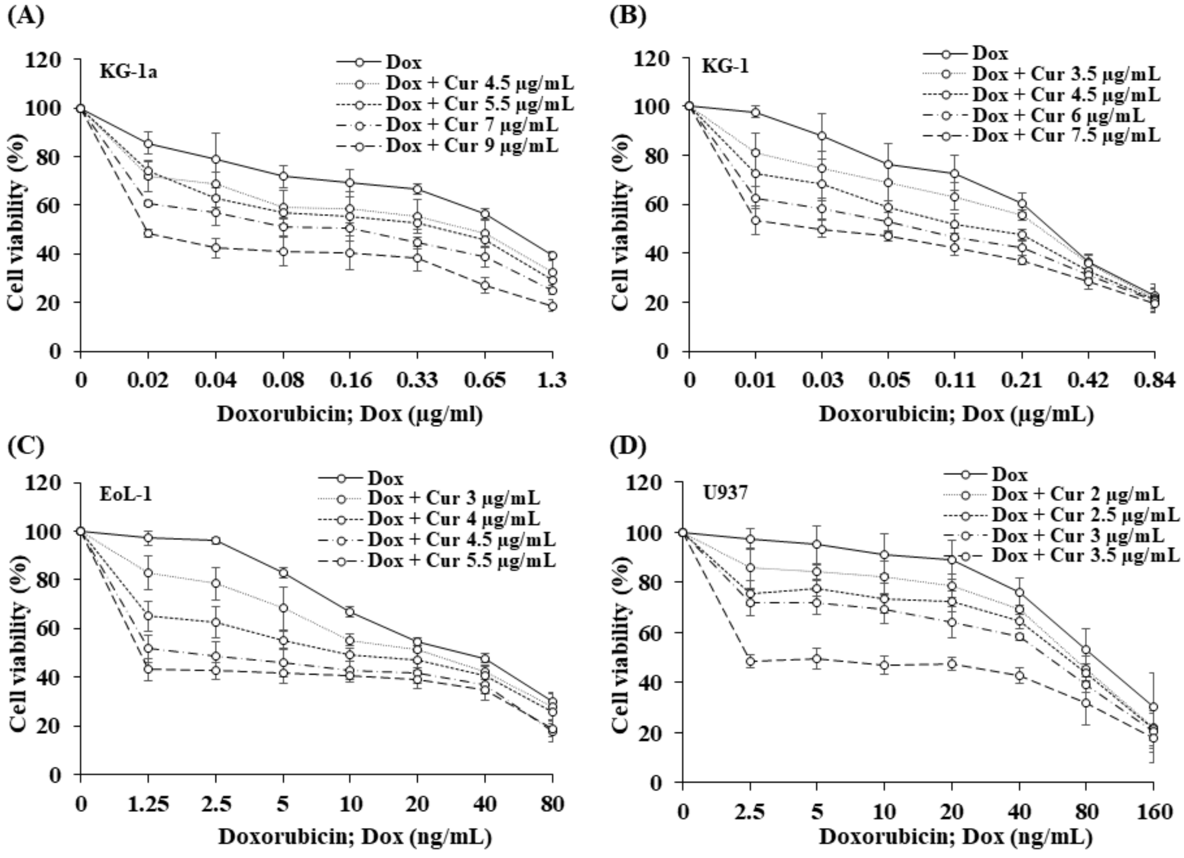

Figure S1. Cytotoxic effects of co-treatment of doxorubicin and curcumin (Dox–Cur) on KG-1a, KG-1, EoL-1, and U937 cell lines. The data are shown as mean ± SD from 3-time independent experiments.

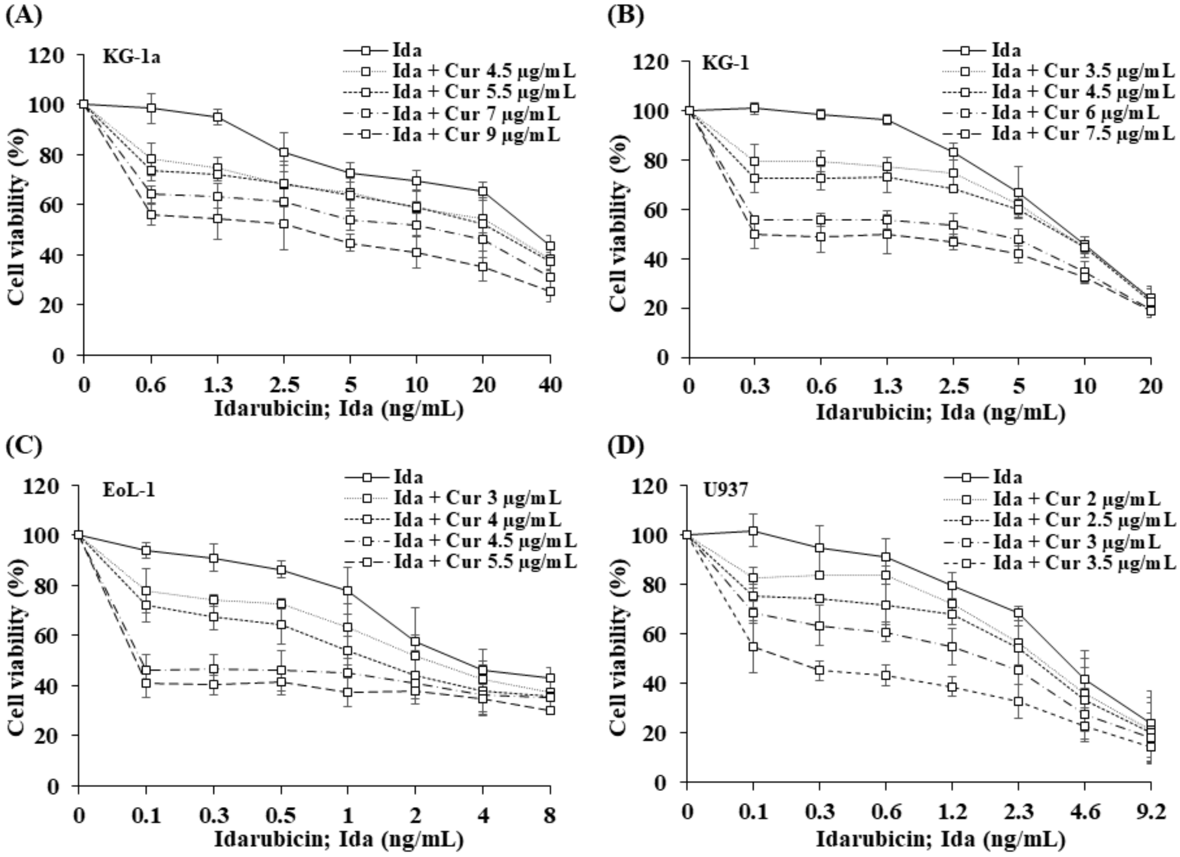

Figure S2. Cytotoxic effects of co-treatment of Idarubicin and curcumin (Ida–Cur) on KG-1a, KG-1, EoL-1, and U937 cell lines. The data are shown as mean ± SD from 3-time independent experiments.

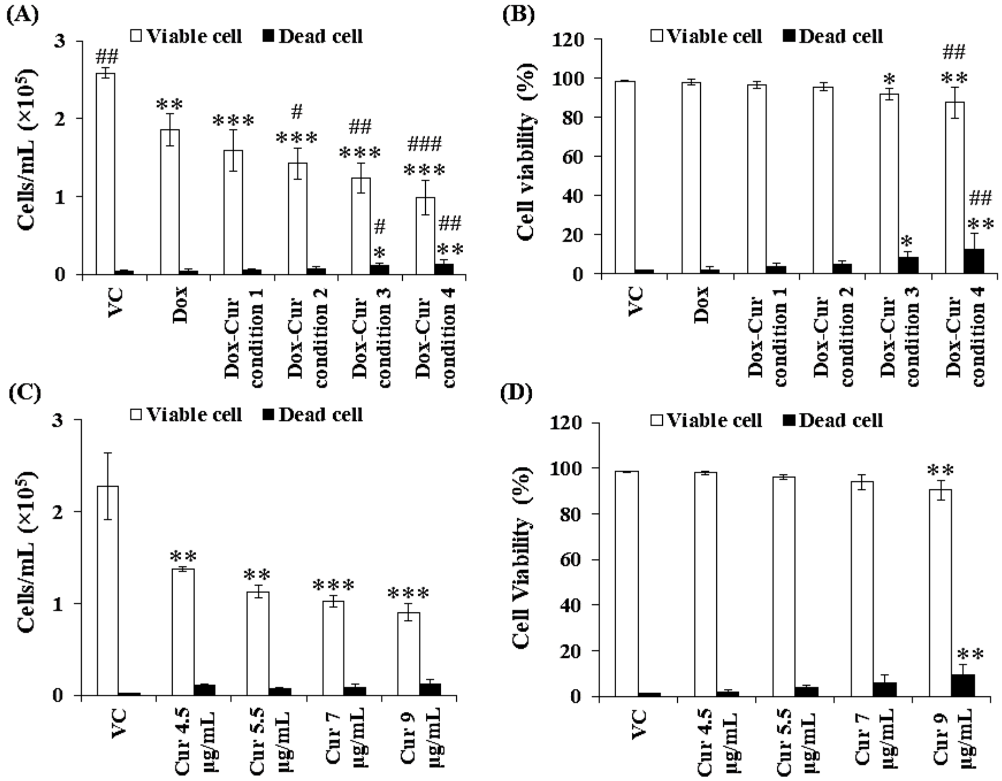

Figure S3. Cell number and cell viability of KG-1a cells after treatment with Dox, Cur, and combined treatment of Dox–Cur for 48 h. (A) Cell number and (B) cell viability of KG-1a cells treated with DMSO (VC), Dox at concentration of 60 ng/mL, Dox–Cur condition 1 (Dox 15 ng/mL + Cur 4.5 µg/mL), Dox–Cur condition 2 (Dox 16 ng/mL + Cur 5.5 µg/mL), Dox–Cur condition 3 (Dox 12 ng/mL + Cur 7 µg/mL), and Dox–Cur condition 4 (Dox 8 ng/mL + Cur 9 µg/mL) for 48 h. (C) Cell number and (D) cell viability of KG-1a cells treated with concentration of DMSO (VC) and various concentrations of Cur at IC_{20} (4.5 µg/mL), IC_{30} (5.5 µg/mL), IC_{40} (7 µg/mL), and IC_{50} (9 µg/mL) for 48 h. The data are shown as mean ± SD from 3 independent experiments. The significance of mean differences was assessed using one-way ANOVA. $^{*}p < 0.05$, $^{**}p < 0.01$, and $^{***}p < 0.001$ compared to VC. $^{\#}p < 0.05$, $^{\#\#}p < 0.01$, and $^{\#\#\#}p < 0.001$ compared to single Dox treatment.

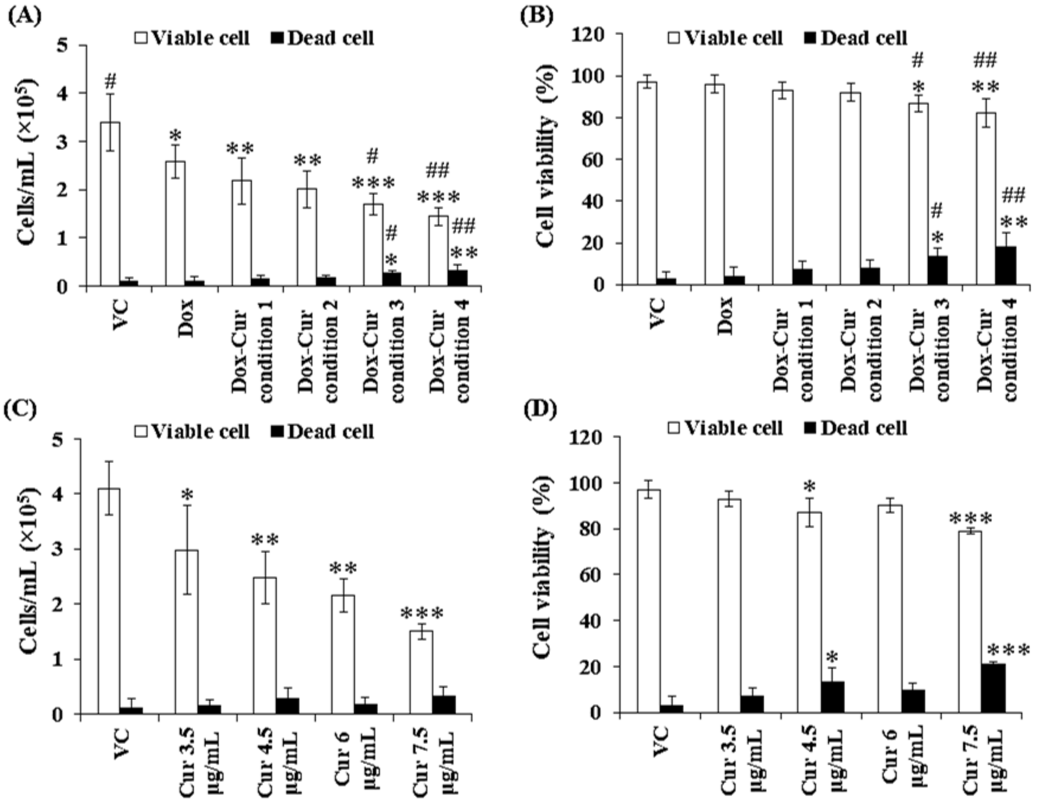

Figure S4. Cell number and cell viability of KG-1 cells after treatment with Dox, Cur, and combined treatment of Dox–Cur for 48h. (A) Cell number and (B) cell viability of KG-1 cells treated with DMSO (VC), Dox at concentration of 58 ng/mL, Dox–Cur condition 1 (Dox 22 ng/mL + Cur 3.5 μg/mL), Dox–Cur condition 2 (Dox 10 ng/mL + Cur 4.5 μg/mL), Dox–Cur condition 3 (Dox 7 ng/mL + Cur 6 μg/mL), and Dox-Cur condition 4 (Dox 6 ng/mL + Cur 7.5 μg/mL) for 48 h. (C) Cell number and (D) cell viability of KG-1 cells treated with concentration of DMSO (VC) and various concentrations of Cur at IC_{20} (3.5 μg/mL), IC_{30} (4.5 μg/mL), IC_{40} (6 μg/mL), and IC_{50} (7.5 μg/mL) for 48 h. The data are shown as mean ± SD from 3 independent experiments. The significance of differences between means was assessed using one-way ANOVA. The significance of mean differences was assessed using one-way ANOVA. $^{*}p < 0.05$, $^{**}p < 0.01$, and $^{***}p < 0.001$ compared to VC. $^{\#}p < 0.05$, $^{\#\#}p < 0.01$, and $^{\#\#\#}p < 0.001$ compared to single Dox treatment.

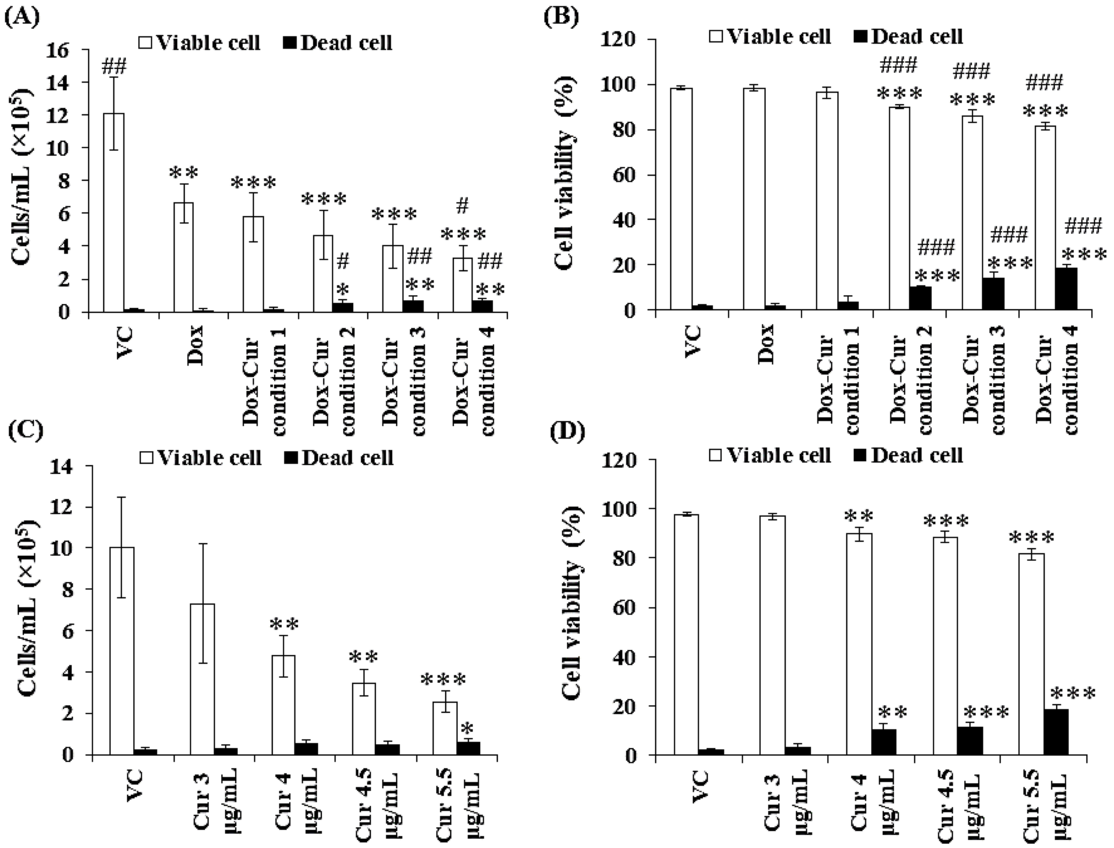

Figure S5. Cell number and cell viability of EoL-1 cells after treatment with Dox, Cur, and combined treatment of Dox–Cur for 48 h. (A) Cell number and (B) cell viability of EoL-1 cells treated with DMSO (VC), Dox at concentration of 6 ng/mL, Dox–Cur condition 1 (Dox 3 ng/mL + Cur 3 µg/mL), Dox–Cur condition 2 (Dox 0.7 ng/mL + Cur 4 µg/mL), Dox–Cur condition 3 (Dox 0.5 ng/mL + Cur 4.5 µg/mL), and Dox–Cur condition 4 (Dox 0.4 ng/mL + Cur 5.5 µg/mL), respectively, for 48 h. (C) Cell number and (D) cell viability of EoL-1 cells treated with concentration of DMSO (VC) and various concentrations of Cur at IC_{20} (3 µg/mL), IC_{30} (4 µg/mL), IC_{40} (4.5 µg/mL), and IC_{50} (5.5 µg/mL) for 48 h. The data are shown as mean ± SD from 3 independent experiments. The significance of differences between means was assessed using one-way ANOVA. $^{*}p < 0.05$, $^{**}p < 0.01$, and $^{***}p < 0.001$ compared to VC. $^{\#}p < 0.05$, $^{\#\#}p < 0.01$, and $^{\#\#\#}p < 0.001$ compared to single Dox treatment.

Supplementary Table

Table S1. IC_{50} values of single and co-treatment of Dox or Ida and Cur on KG-1a cells.

**Treatment (combination with)**	**IC_{50} value**	
	**Dox (μg/mL)**	**Ida (ng/mL)**
+ Vehicle control	0.89 ± 0.07	33.29 ± 5.02
+ Cur (4.5 μg/mL) (condition 1)	0.52 ± 0.22**	21.86 ± 9.19
+ Cur (5.5 μg/mL) (condition 2)	0.45 ± 0.14**	21.89 ± 4.98
+ Cur (7.0 μg/mL) (condition 3)	0.17 ± 0.10***	14.25 ± 8.87**
+ Cur (9.0 μg/mL) (condition 4)	0.02 ± 0.01***	3.15 ± 1.97***

The significance of mean differences was assessed using one-way ANOVA. *$p < 0.05$, **$p < 0.01$, and ***$p < 0.001$ compared to single drug treatment.

Table S2. IC_{50} values of single and co-treatment of Dox or Ida and Cur on KG-1 cells.

**Treatment (combination with)**	**IC_{50} value**	
	**Dox (μg/mL)**	**Ida (ng/mL)**
+ Vehicle control	0.30 ± 0.01	8.68 ± 1.29
+ Cur (3.5 μg/mL) (condition 1)	0.27 ± 0.01	8.47 ± 1.23
+ Cur (4.5 μg/mL) (condition 2)	0.14 ± 0.08***	8.22 ± 1.08
+ Cur (6.0 μg/mL) (condition 3)	0.07 ± 0.03***	4.21 ± 1.59**
+ Cur (7.5 μg/mL) (condition 4)	0.02 ± 0.01***	0.96 ± 1.11***

The significance of mean differences was assessed using one-way ANOVA. *$p < 0.05$, **$p < 0.01$, and ***$p < 0.001$ compared to single drug treatment.

Table S3. IC_{50} values of single and co-treatment of Dox or Ida and Cur on EoL-1 cells.

**Treatment (combination with)**	**IC_{50} value**	
	**Dox (μg/mL)**	**Ida (ng/mL)**
+ Vehicle control	33.29 ± 4.80	3.66 ± 2.14
+ Cur (3.0 μg/mL) (condition 1)	22.77 ± 2.66*	2.65 ± 1.17
+ Cur (4.0 μg/mL) (condition 2)	13.16 ± 7.16***	1.89 ± 1.30
+ Cur (4.5 μg/mL) (condition 3)	3.23 ± 2.35***	0.12 ± 0.01**
+ Cur (5.5 μg/mL) (condition 4)	1.10 ± 0.09***	0.11 ± 0.01**

The significance of mean differences was assessed using one-way ANOVA. *$p < 0.05$, **$p < 0.01$, and ***$p < 0.001$ compared to single drug treatment.

Table S4. IC_{50} values of single and co-treatment of Dox or Ida and Cur on U937 cells.

Treatment (combination with)	IC_{50} value	
	**Dox (μg/mL)**	**Ida (ng/mL)**
+ Vehicle control	96.18 ± 24.25	4.37 ± 1.24
+ Cur (2.0 μg/mL) (condition 1)	72.92 ± 6.58*	3.40 ± 1.39
+ Cur (2.5 μg/mL) (condition 2)	68.76 ± 4.91*	3.01 ± 1.07
+ Cur (3.0 μg/mL) (condition 3)	57.60 ± 3.78**	1.76 ± 0.94*
+ Cur (3.5 μg/mL) (condition 4)	2.44 ± 0.11***	0.18 ± 0.07**

The significance of mean differences was assessed using one-way ANOVA. *$p < 0.05$, **$p < 0.01$, and ***$p < 0.001$ compared to single drug treatment.
